# *Bordetella pertussis* Autotransporter Vag8 Binds Human C1 Esterase Inhibitor and Confers Serum Resistance

**DOI:** 10.1371/journal.pone.0020585

**Published:** 2011-06-14

**Authors:** Nico Marr, Nita R. Shah, Rose Lee, Emma J. Kim, Rachel C. Fernandez

**Affiliations:** Department of Microbiology & Immunology, University of British Columbia, Vancouver, British Columbia, Canada; University of California Los Angeles, United States of America

## Abstract

*Bordetella pertussis* employs numerous strategies to evade the immune system, including the ability to resist killing via complement. Previously we have shown that *B. pertussis* binds a complement regulatory protein, C1 esterase inhibitor (C1inh) to its surface in a Bvg-regulated manner (*i.e.* during its virulence phase), but the *B. pertussis* factor was not identified. Here we set out to identify the *B. pertussis* C1inh-binding factor. Using a serum overlay assay, we found that this factor migrates at approximately 100 kDa on an SDS-PAGE gel. To identify this factor, we isolated proteins of approximately 100 kDa from wild type strain BP338 and from BP347, an isogenic Bvg mutant that does not bind C1inh. Using mass spectrometry and bioinformatics, we identified the autotransporter protein Vag8 as the putative C1inh binding protein. To prove that Vag8 binds C1inh, *vag8* was disrupted in two different *B. pertussis* strains, namely BP338 and 18–323, and the mutants were tested for their ability to bind C1inh in a surface-binding assay. Neither mutant strain was capable of binding C1inh, whereas a complemented strain successfully bound C1inh. In addition, the passenger domain of Vag8 was expressed and purified as a histidine-tagged fusion protein and tested for C1inh-binding in an ELISA assay. Whereas the purified Vag8 passenger bound C1inh, the passenger domain of BrkA (a related autotransporter protein) failed to do so. Finally, serum assays were conducted to compare wild type and *vag8* mutants. We determined that *vag8* mutants from both strains were more susceptible to killing compared to their isogenic wild type counterparts. In conclusion, we have discovered a novel role for the previously uncharacterized protein Vag8 in the immune evasion of *B. pertussis*. Vag8 binds C1inh to the surface of the bacterium and confers serum resistance.

## Introduction

Complement is a critical and multifaceted host defense system comprised of a series of proteins—many of which are zymogens—found largely in plasma [1] but also on respiratory mucosal surfaces [2], [3]. Three activation pathways of complement are known today, designated the classical, lectin, and alternative pathway. Following activation, complement components of all three pathways act in a defined cascade to effect immune clearance of microorganisms either directly by assembly of membrane attack complexes, or indirectly by opsonization and release of peptide mediators of inflammation which promote the attraction of and recognition by various immune cells [1]. To protect the host from the effector functions of complement and prevent rapid consumption of complement components in response to trivial stimuli, all three activation pathways of complement are tightly regulated at different stages by a number of complement regulatory proteins [4]. *Bordetella pertussis*, the causative agent of whooping cough, has been shown to express a variety of virulence-associated factors that in concert enable the bacteria to colonize the mucosa of the upper respiratory tract in humans. Expression of most virulence-associated factors in *B. pertussis* is positively regulated by the BvgAS two component system [5], [6]. Apart from adherence of the bacteria to the ciliated respiratory epithelium, many of these factors help the bacteria to evade or modulate host immune defenses [7], [8]. One of the immune defenses to overcome is complement.

There is a basic necessity for *B. pertussis* to prevent complement activation because the *B. pertussis* endotoxin lacks a repetitive O polysaccharide [9] rendering the bacteria particularly vulnerable to direct, complement-mediated bacterial lysis—unlike many other Gram-negative bacteria including other *Bordetella* species where endotoxin can act as a protective shield [10]. Indeed, *B. pertussis* is relatively resistant to killing by complement [11]. We have previously shown that the *B. pertussis* autotransporter protein BrkA mediates resistance to the classical complement pathway [11] and although this happens at a very early stage in the pathway [12], the underlying molecular mechanism remains elusive. Interestingly, *B. pertussis* expresses other factors in its virulence phase that can interact with complement components or its regulators, either directly or indirectly. Filamentous hemagglutinin (FHA) has been shown to bind a complement regulator called C4-binding protein (C4BP) [13], [14], however how this affects serum resistance is not known since *B. pertussis* mutants deficient in FHA expression exhibit a level of complement resistance that is not significantly different compared to their parental wild type strain [15]. We have recently demonstrated that *B. pertussis* is able to recruit another complement regulatory protein of humans, C1 esterase inhibitor (C1inh). This phenotype is associated with resistance to complement-mediated killing but requires neither the expression of BrkA nor FHA [16], [17]. With the present study we set out to identify the *B. pertussis* factor that is responsible for the binding of human C1inh to the bacterial surface, hence also crucial for serum resistance. This factor was found to be the autotransporter protein Vag8.

## Results

### Identification of the B. pertussis factor that mediates recruitment of human C1-esterase inhibitor

We have previously shown that *B. pertussis* is capable of binding human C1inh to its surface and that this is dependent on signal transduction by the BvgAS two-component system, the master virulence regulatory system of *Bordetella* species. Furthermore, the ability of *B. pertussis* to bind human C1inh was found to be independent of the Bvg-activated serum resistance protein BrkA [16]. In the present study we aimed to identify the bacterial ligand that interacts with human C1inh. We first tested a variety of well-characterized *B. pertussis* mutants with defects in BvgAS-activated genes using the C1inh binding assay that we described previously [16]. These mutants included: BBC9BrkA (a derivative of wild type W28) that does not express pertactin and BrkA [11], [18]; SK34 (a derivative of wild type 18–323) that harbors a Tn*phoA* insertion in the *tcfA* locus [19]; BP338 (Tohama I)-derived Tn*5lac* mutants BPM3171 and BPM1809 which fail to secrete pertussis toxin [20] and lack the expression of dermonecrotic toxin [21], respectively; as well as BP338 (Tohama I)-derived mutants BP348 and BP353 which harbor Tn*5* insertions in *cyaA* (adenylate cyclase toxin) and *fimC* (fimbria), respectively. The latter strain also does not express filamentous haemagglutinin (FHA) due to a polar effect of the transposon insertion [22], [23]. Wild type BP338 and the isogenic BvgS mutant BP347 (*bvgS*::Tn*5*) [22], [23] were used as positive and negative controls, respectively. We found that with the exception of the BvgS mutant BP347, none of the *B. pertussis* mutants tested here was visibly compromised in C1inh binding (data not shown).

**Figure 1:**
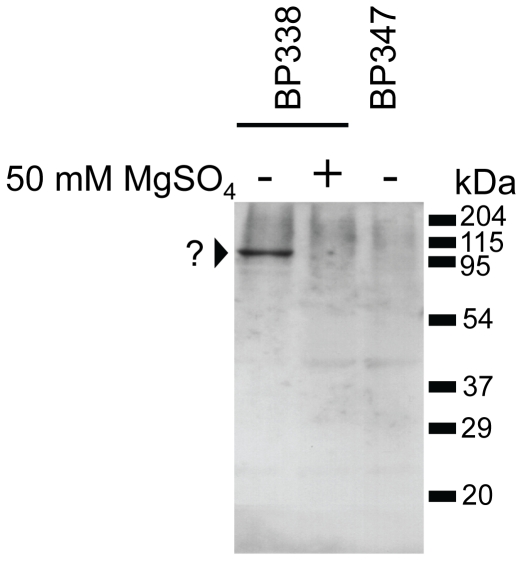
Far-Western analysis showing interaction of C1inh from normal human serum with an unknown *B. pertussis* factor migrating at ca. 90 to 100 kDa. *B. pertussis* strain BP338 (wild type) and its isogenic mutant BP347 (*bvgS*::Tn*5*) were grown without (−) or with (+) Bvg-modulating agent MgSO_4_ (50 mM) prior to performing the Far-Western assay using normal human serum as the probe, followed by detection using anti-C1inh antibody.

Using microarray-based transcriptional profiling of Bvg^+^ and Bvg^−^ phase-locked mutants, Cummings et al. [5] revealed that 155 genes are Bvg-activated in *B. pertussis* Tohama I-derived strains. To narrow down the list of candidate *B. pertussis* factors potentially involved in the recruitment of C1inh, we employed a modification of a so-called Far-Western or gel-overlay strategy described elsewhere [24], in combination with mass spectrometry. This technique has been used to study protein-protein interactions [24] and it enabled us to determine the approximate molecular mass of the factor expressed by *B. pertussis* that mediates recruitment of C1inh. In brief, whole cell lysates of *B. pertussis* strains BP338 and BP347 were subjected to SDS-PAGE. Separated proteins were blotted onto a PVDF membrane and subsequently renatured. Membranes were then overlaid with 1% normal human serum, and human C1inh interacting with any *B. pertussis* factor on the PVDF membrane was detected by standard immunoblot analysis. Using this approach, we found human C1inh to interact with a *B. pertussis* factor migrating at approximately 95 to 100 kDa that was expressed in wild type BP338 but absent in BvgS mutant BP347 (Fig. 1). To validate that the bacterial factor in question is indeed subject to BvgAS regulation, we cultivated wild type BP338 in the absence or presence of 50 mM MgSO_4_ (which turns off the Bvg system) and employed the Far-Western strategy described above. As shown in figure 1, the *B. pertussis* factor capable of binding human C1inh was absent when the bacteria were grown under BvgAS-modulating conditions.

To identify BvgAS-regulated bacterial factors migrating at ca. 95 to 100 kDa on SDS-PAGE gels, crude membrane enrichments of wild type strain BP338 (grown under Bvg-activating conditions) and BvgS mutant BP347 were separated by SDS-PAGE and the polyacrylamide gel stained with Coomassie blue. A visible protein band migrating at the appropriate molecular weight and present in BP338 but absent in BP347 was excised out of the protein gel. Corresponding regions from the lane containing membrane enrichments of BvgS mutant BP347 were similarly excised. Trypsin-digested peptides of the gel samples from both strains were subjected to MS/MS analysis, and a search of the obtained data against the protein sequence database MSBD was performed using Mascot. Using this approach, we identified five significant matches of proteins that were present in membrane enrichments of BP338, but were not detected in BvgS mutant BP347; namely the gene product of virulence-activated gene 8 (Vag8), Bvg-intermediate phase protein A (BipA), pertactin, a pyruvate dehydrogenase E1 component and DNA gyrase subunit B (Table 1).

**Table 1:** Genes encoding the proteins that were identified by tandem mass spectrometry.

	Gene name	Gene product	Probability Based Mowse Score	
			BP338	BP347
BP2315	*vag8*	autotransporter Vag8	2011	not detected
BP2014	*acnA*	Putative aconitate hydratase	1265	1251
BP1112	*bipA*	putative OM ligand binding protein (BipA)	1185	not detected
BP0197	*gcvP*	glycine cleavage system P protein	not detected	238
BP2497		putative zinc protease	781	202
BP0460	*topB*	DNA topoisomerase iii	346	458
BP0869	*pepN*	aminopeptidase N	240	621
BP0993	*aceE*	pyruvate dehydrogenase E1 component	234	not detected
BP1836	*alaS*, *lovB*	alanyl-tRNA synthetase	219	662
BP0489	*gyrB*	DNA gyrase subunit B	116	not detected
BP2573	*pheT*	phenylalanyl-tRNA synthetase beta chain PheT	76	527
BP0944	*gyrA*	DNA gyrase subunit A	67	273
BP2044		leucyl-tRNA synthetase	58	565
BP3467		putative exported protein	56	129
BP1054	*prn*	pertactin	54	not detected
BP1549	*dnaX*	DNA polymerase III subunit Tau	not detected	168
BP1566	*mutS*	DNA mismatch repair protein mutS	not detected	143
BP2184	rpoD	RNA polymerase sigma factor	not detected	130
BP1198	*clpB*, *htpM*	protease ClpB, ATPase subunit	not detected	67
BP1417	*glnD*	[Protein-PII] uridylyltransferase	not detected	66

(MS/MS) analysis and ions search against protein sequence database MSDS using Mascott. All significant hits (*P*<0.05) with individual ions scores >50 are listed, from highest score (top) to lowest (bottom).

**Figure 2:**
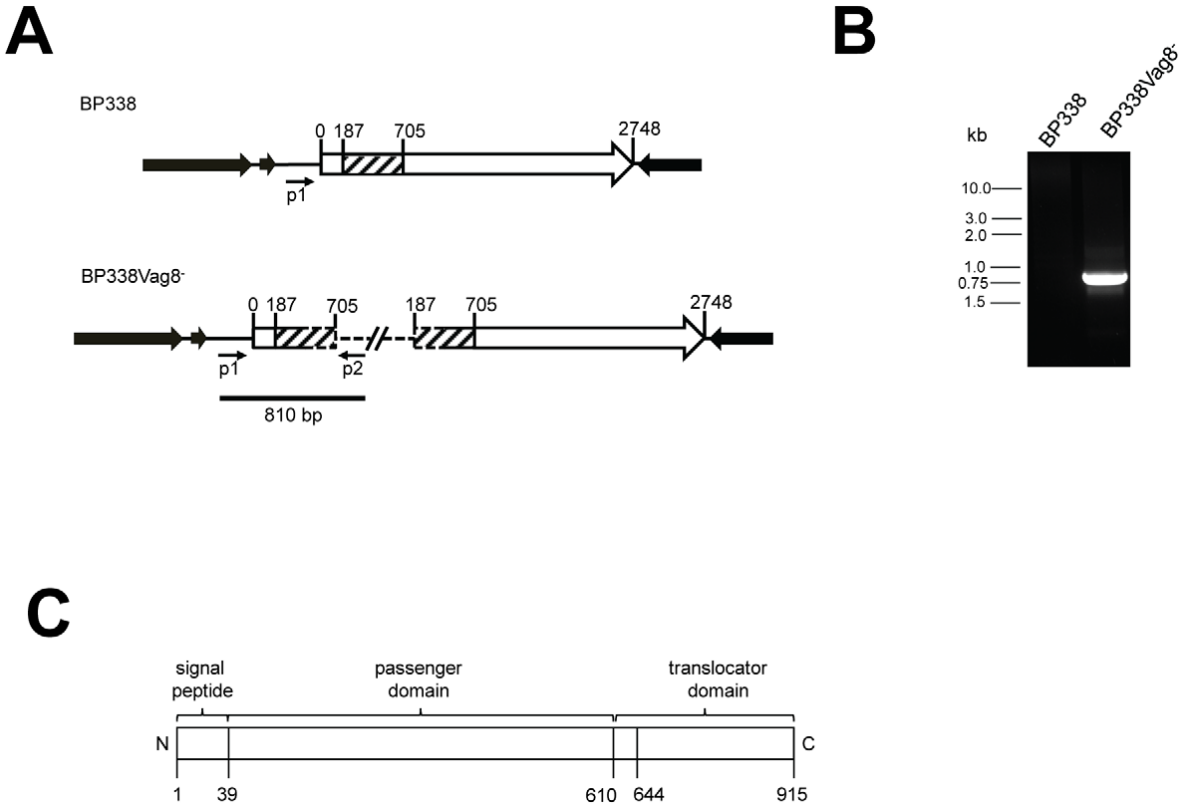
Generation of *vag8* mutants and description of the vag8 genetic locus and predicted protein architecture. **A** Schematic of the *vag8* locus that is knocked out in *B. pertussis* mutant BP338Vag8^−^. The 2748-bp coding sequence of *vag8* is shown as an open arrow, with the hatched region indicating the internal *vag8* fragment which was cloned into vector pEG7 to generate vector pEG7-Vag8. Dashed lines indicate vector sequence in the mutant strain. Solid lines indicate genomic sequence of BP338. *vag8* is flanked by rRNA genes at the 5′ end and locus tag BP2314 at the 3′ end (solid arrows, not drawn to scale). The bar indicates the 810-bp PCR product (shown in **B**) that was indicative of the correct genomic integration of pEG7-Vag8 into the *vag8* gene. **C** Architecture of the 95-kDaVag8 autotransporter protein. The predicted signal peptide and the passenger and translocator domains are indicated. The region spanning residues 610 and 644 comprise a predicted alpha-helical linker region present in all autotransporter proteins. Unlike many autotransporter proteins, Vag8 is not cleaved to separate its passenger domain from the translocator. The duplicated fragments of *vag8* in BP338Vag8^−^ are missing sequences encoding the translocator and signal peptide respectively.

Because the latter two hits are components of essential cytosolic enzymes that were likely present in BvgS mutant BP347 but which remained undetected, and since the *B. pertussis* pertactin-BrkA double mutant BBC9BrkA was found to be capable of binding C1inh, we decided to focus on Vag8 and BipA. *bipA* and *vag8* were disrupted using suicide vectors pTEN34 and pEG7-vag8, respectively and hemolytic *B. pertussis* mutants with genomic insertions of pTEN34 or pEG7-vag8 were isolated and designated as BP338BipA^−^ or BP338Vag8^−^. Correct genomic integration of the suicide vectors was validated by PCR of the genomic insertion site using vector-specific primers in combination with a *bipA* or a *vag8* promoter-specific primer that could not hybridize to pTEN34 or pEG7-vag8, respectively. PCR products of the expected sizes were found when using genomic DNA of both mutants as templates, whereas no product was detected with genomic DNA of wild type BP338 that served as the negative control. The *vag8* results are shown in Figure 2(A and B). The PCR products generated from genomic DNA of mutants BP338BipA^−^ and BP338Vag8^−^ were sequenced and found to encode the expected genomic insertion sites.

**Figure 3:**
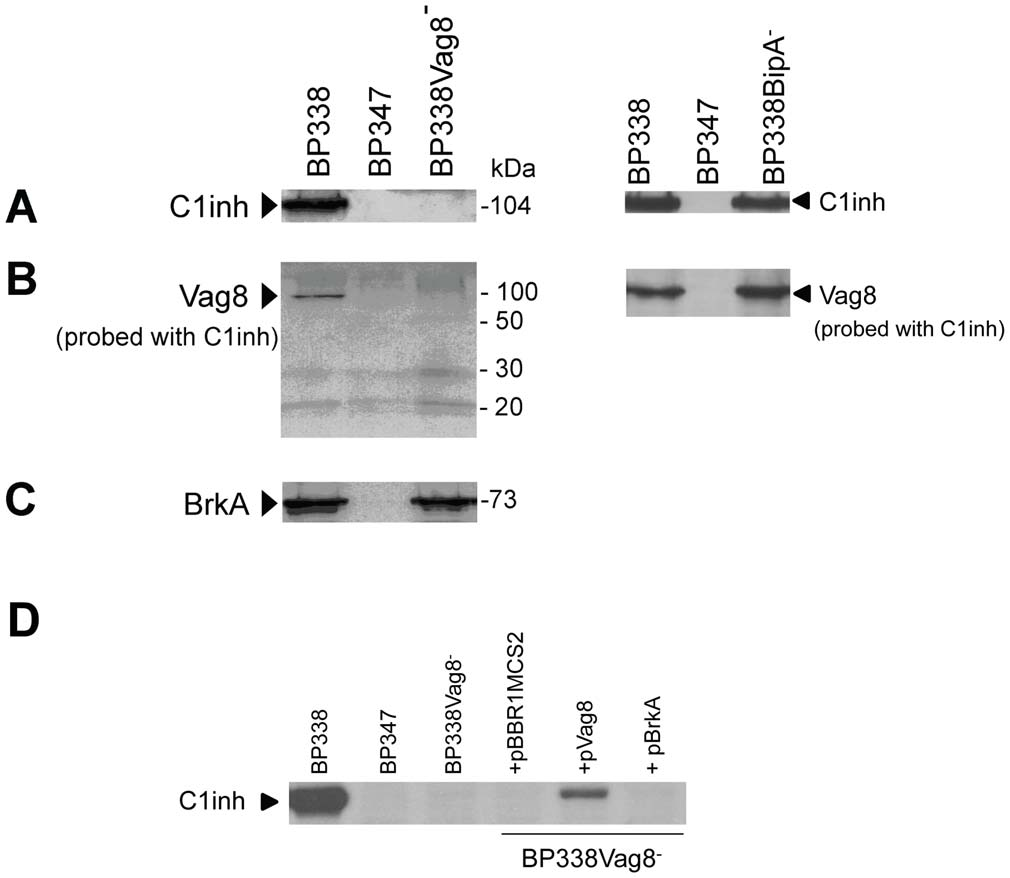
A *B. pertussis* Vag8 mutant fails to bind C1inh. **A** Standard immunoblot analysis showing surface binding of the 104-kDa C1inh protein to whole bacteria incubated in serum; **B** Far-Western analysis testing for the interaction of C1inh with a ca. 95- to 100-kDa factor of *B. pertussis*. The left and right panels in **A** and **B** show the results with BP338Vag8^−^ (*vag8*::pEG7-Vag8) and BP338BipA^−^ (*bipA*::pTEN34), respectively. **C** Immunoblot probed for the BvgAS-activated autotransporter protein BrkA. **D** Immunoblot analysis showing surface binding of C1inh to wild type *B. pertussis* (BP338) and the BP338Vag8^−^ mutant complemented with pVag8. BP338Vag8^−^ complemented with control plasmids pBBR1MCS2 (empty vector) or pBrkA (vector with the *brkA* gene under control of P*_cpn10_*) did not bind C1inh.

Following verification of the mutation in BP338BipA^−^ and BP338Vag8^−^, the two *B. pertussis* mutants were then assayed for their ability to recruit human C1inh from human serum [16] and to bind to C1inh by use of the Far-Western approach described above. Wild type strain BP338 and BvgS mutant BP347 served as positive and negative controls, respectively. Whereas *B. pertussis* mutant BP338BipA^−^ was found to be capable of binding human C1inh using both the surface-binding and the Far Western overlay assays, BP338Vag8^−^ was not (Fig. 3A and B). The lack of C1inh binding was not due to phase variation of the Bvg system as demonstrated by the expression of the Bvg-activated BrkA protein (Fig. 3C), and by hemolysis which is a function of the Bvg-activated adenylate cyclase toxin. Furthermore, it is unlikely that the lack of C1inh binding was due to a polar effect of the insertion mutation as there are no genes in the same orientation immediately downstream of *vag8*, and the gene immediately following *vag8* is in the opposite orientation (Fig. 2A). In any event, to reproduce these findings, independent Vag8 mutants were generated again in BP338 and in a streptomycin-resistant derivative of a different *B. pertussis* strain called 18–323. All of the Vag8 mutants expressed BrkA and were hemolytic, but were unable to bind C1inh. These results strongly implicate Vag8 as the C1inh-binding factor. Finally, we complemented one of the Vag8 mutants originated from *B. pertussis* BP338 with pVag8, a plasmid that replicates in bordetellae and encodes a full-length open reading frame (ORF) of *vag8* driven by promoter P*_cpn10_*. In parallel, we introduced an empty vector control (pBBR1MCS2) or pBrkA (a similar plasmid encoding *brkA* instead of *vag8*) into this Vag8 mutant via conjugation. As shown in Fig. 3D, we were able to partially restore the ability to bind C1inh in the Vag8 mutant complemented with the plasmid that encodes a full-length *vag8* ORF, whereas no C1inh could be observed in the conjugants harboring the empty vector control or pBrkA.

**Figure 4:**
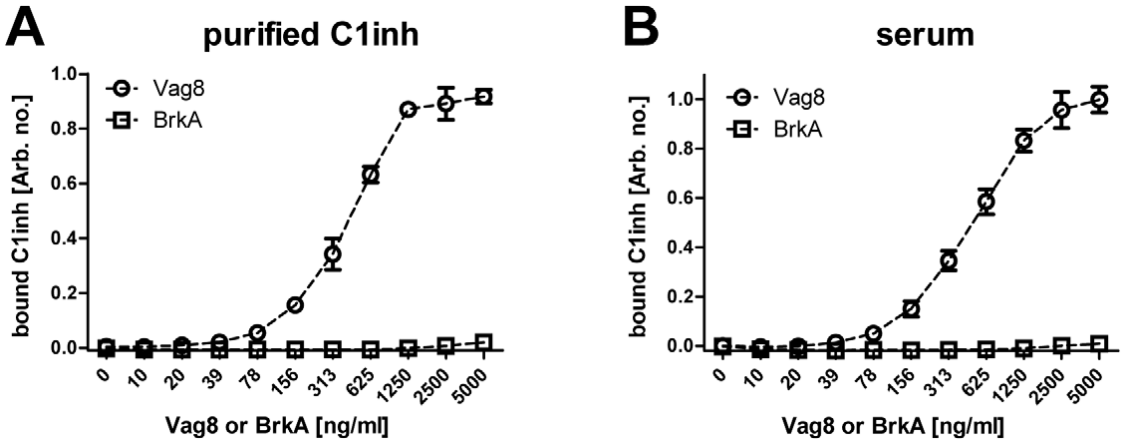
Purified histidine-tagged Vag8 passenger domain binds C1inh. Shown are means ± SD of a representative ELISA done in triplicate, where Nunc MaxiSorp® flat-bottom 96 well plates were coated with increasing two-fold concentrations of purified histidine-tagged Vag8 passenger or purified histidine-tagged BrkA passenger, and after blocking non-specific binding, incubated with either 10 µg/ml purified C1inh (**A**), or 2% normal human serum diluted in 1× Veronal buffer (**B**). For each graph shown, two independent experiments were done.

Vag8 is an autotransporter protein [25]. As is typical of autotransporter proteins, it has a surface-exposed passenger domain that provides the effector function, and a translocator domain that is involved in secretion and anchoring of the Vag8 passenger to the bacterial surface (Fig. 2C). To further validate that Vag8 binds C1inh, the passenger domain of Vag8 comprised of residues Val^40^ to Leu^610^ was fused to a histidine tag and purified using nickel chromatography. An ELISA was used to examine whether the purified Vag8 passenger is capable of directly binding C1inh. Briefly, Immuno 96 MicroWell MaxiSorp™ plates were coated with different concentrations of the purified Vag8 passenger domain, and after washing and then blocking non-specific binding, the plates were probed with either purified C1inh or human serum. Bound C1inh was detected using anti-C1inh antiserum. Results using this assay confirm direct binding of the Vag8 passenger domain to purified C1inh (Fig. 4A) or C1inh from human serum (Fig. 4B) in a dose-dependent manner. In contrast, the similar-sized (73-kDa) BrkA passenger domain [26] which shares 27% identity to Vag8 (residues 205–604) failed to bind C1inh. Similar observations were made using the Far-Western approach described above (data not shown). No signal was detected when we performed the ELISA on wells that were coated with 5 µg/ml of the purified Vag8 passenger domain but not probed with purified C1inh or serum, ruling out non-specific binding of the antibodies to Vag8. Taken together, our data show that the Vag8 passenger domain binds directly to human C1inh.

### Serum sensitivity in the absence of Vag8 expression

**Figure 5:**
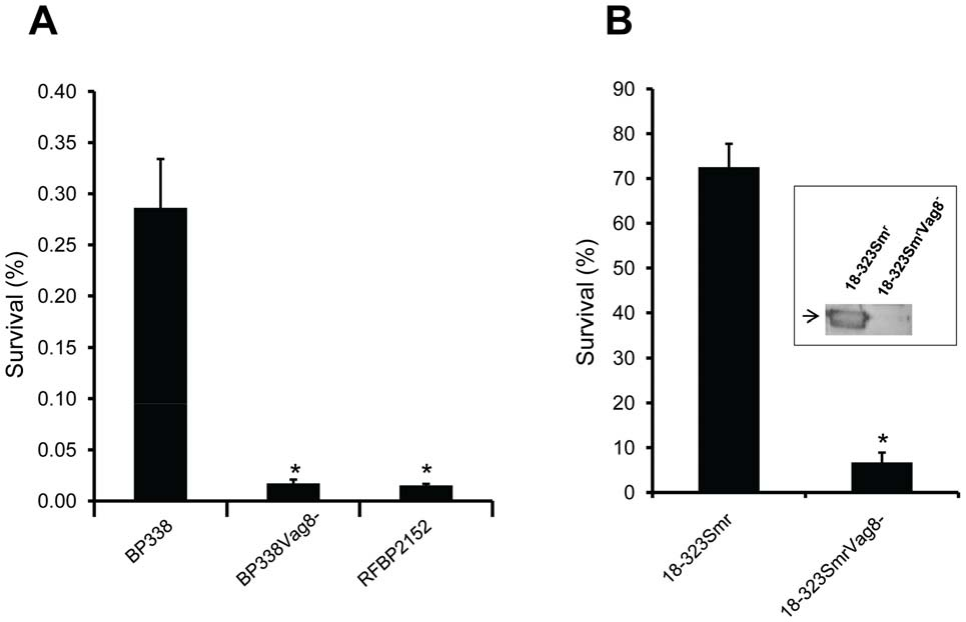
Results of serum killing assays. **A** *B. pertussis* BP338 and its isogenic mutants, RFBP2152 (*brkA*::*gent*) and BP338Vag^−^ (*vag8*::pEG7-Vag8). Shown are means of percent survival ± SEM of a representative experiment done in triplicate, *, *P*<0.04 vs. wild type strain. The comparison of BP338 and BP338Vag8^−^ was done 4 times independently, in triplicate; three of those experiments included RFBP2152. **B** *B. pertussis* 18–323Sm^r^ and its isogenic mutant 18–323Sm^r^Vag8^−^ (*vag8*::pEG7-Vag8). Shown are means of percent survival ± SEM of a representative experiment done twice in triplicate. *, *P*<0.004. Inset shows binding of C1inh (arrow) to wild type 18–323Sm^r^ but not to 18–323Sm^r^Vag8^−^.

We have previously shown that the ability of *B. pertussis* to bind human C1inh is well-correlated with serum resistance [16]. By using a serum killing assay, we aimed to confirm that expression of Vag8 by *B. pertussis*, which we have shown to be essential for binding human C1inh, is also crucial for serum resistance. We first assayed BP338Vag8^−^ and its parental strain BP338, as well as BrkA mutant RFBP2152. As shown in figure 5A, mutant BP338Vag8^−^ exhibited similar serum sensitivity levels as the BrkA mutant RFBP2152; both were significantly more sensitive to serum-mediated killing than their parental wild type strain BP338. We next looked at 18–323Sm^r^Vag8^−^ and its parental strain 18–323Sm^r^. In comparison to its parental strain, 18–323Sm^r^Vag8^−^ was also unable to bind C1inh and was significantly more serum sensitive (Fig. 5B). Finally, we assessed serum resistance in the BP338Vag8^−^ strain complemented with pVag8. The partial complementation of C1inh-binding exhibited in this strain was not sufficient to restore serum resistance (not shown). In this regard, it should be noted that whereas the BP338 and BP338Vag8^−^ strains grow similarly, the complemented strain grows very slowly – likely due to the selection pressure imposed by 2 antibiotics, further complicating efforts to compare serum resistance using this strain.

## Discussion

Vag8 is a Bvg-activated 95-kDa autotransporter protein of *B. pertussis* [25], [27] whose function has remained elusive [28]. In the present study we showed that disruption of *vag8* abolishes binding of C1inh and that complementing a Vag8 mutant with a plasmid encoding a full-length *vag8* ORF under control of the non-native promoter P*_cpn10_* partially restores the C1inh-binding phenotype. Furthermore, we demonstrated that purified Vag8 protein (passenger domain) but not BrkA binds to C1inh in a dose-dependent manner. These experiments clearly indicate that Vag8 is the C1inh binding factor. We also showed that disrupting *vag8* (done two times independently in strain BP338, and once in 18–323) significantly diminishes resistance to serum killing, linking the phenotypes of C1inh binding and serum resistance to Vag8 expression. Taken together, our results show that expression of Vag8 is essential for the ability of *B. pertussis* to bind human C1inh during the virulent phase, and confers serum resistance.

Previously, a Tn*PhoA* Vag8 mutant derived from *B. pertussis* strain 18–323 was found to be slightly but not significantly more serum sensitive than its parental strain which suggested a negligible role of Vag8 in complement resistance [15]. However, in that study the parental strain was also found to be significantly more serum sensitive in comparison to BP338 [15], whereas in the present study, using a streptomycin-resistant derivative of 18–323, we found the opposite. It is possible that low levels of serum resistance of *B. pertussis* wild type strain 18–323 reported in the previous study [15] were due to second site mutations in this strain. Barnes and Weiss [29] have shown that growth phase affects susceptibility to complement so it is also possible that the increased sensitivity to killing of 18–323 seen in the previous study may have been due to a growth-phase effect since the growth rates of BP338 and 18–323 and its derivatives are different. Indeed, in addition to growth phase, the absolute levels of serum resistance can be affected by a number of parameters including strain origin, the concentration of bacteria and serum used in the assay, time of exposure to serum, and the labile nature of complement. Nevertheless, despite the variability of the absolute levels of serum resistance between experiments, the relative difference between wild type and Vag8 mutants, in both BP338 and 18–323, was always maintained and found to be approximately 10-fold.

Pathogens have evolved a number of mechanisms to evade the complement system [30], [31], including the ability to cleave complement components or to bind complement regulatory proteins that protect the host against unwanted damage. StcE is a secreted metalloprotease from *Escherichia coli* O157:H7 that binds to and cleaves C1inh, yet protects *E. coli* from complement-dependent lysis [32]. StcE is proposed to bind the cell surface and engage the heavily glycosylated amino terminus of C1inh. The esterases C1r or C1s, which are involved in initiating the complement cascade, are then inactivated by the cell-surface bound C1inh. According to the model, it is after this step that StcE cleaves C1inh to release the inactive C1inh-C1r/s complex allowing it to become available for another round of the cycle. This model has also been proposed for the *Aeromonas hydrophila* ortholog TagA [33]. The mechanism by which Vag8 protects *B. pertussis* is likely to be different. Vag8 shares no sequence identity with StcE and it is not predicted to be a protease. Furthermore, while it is not known how Vag8 engages C1inh, binding of C1inh to *B. pertussis* requires its active serpin domain conformation but not N-linked glycosylation [16] suggesting that how Vag8 interacts with C1inh may also be different.

We have previously found that whereas phylogenetically distinct strains of *B. pertussis* (Tohama I derivative BP338 and 18–323) were capable of binding human C1inh, strains of other *Bordetella* species, including *B. bronchiseptica* RB50 and NCTC 10540, *B. parapertussis* 17903. *B. holmesii* ATCC 51541and *B. avium* Wba70 were not [16]. Accordingly, in contrast to *B. pertussis* Tohama I and 18–323, the Vag8 protein was found elsewhere [28] to be poorly expressed in various *B. bronchiseptica* isolates and was undetectable in whole-cell lysates from *B. parapertussis* isolates (upon growth under similar conditions)—despite the presence of the Vag8 coding sequence in the genome of these *Bordetella* species. In the *B. avium* genome, a *vag8* gene is altogether absent [34]. A *vag8* deletion mutant derived from *B. pertussis* 18–323 was found to colonize mice as efficiently as the parental strain in a mouse aerosol model of *B. pertussis* infection [28]. Thus, given these observations, the role of Vag8-mediated binding of C1inh during *B. pertussis* infection needs further study in order to fully elucidate its role in *B. pertussis* pathogenesis. In this regard, it is interesting to note that, in addition to the complement pathway, C1inh also inhibits bradykinin formation. Bradykinin is a key mediator of cough and it has been postulated that the sequestration of C1inh by *B. pertussis* could result in its depletion in airways, leading to coughing due to increased levels of bradykinin in the airways [35].

*B. pertussis* has a remarkable ability to bind human complement-regulatory proteins. How this contributes to *B. pertussis* pathogenesis is under investigation in our laboratory.

## Materials and Methods

### Bacterial strains, plasmids and growth conditions

*B. pertussis* strains were grown at 37°C on Bordet-Gengou (BG) agar (BD Biosciences) with 15% sheep blood (Dalynn) for 3 to 4 days. *E. coli* strains were cultured at 37°C in Luria-Bertani (LB) broth or on LB agar. The following antibiotics were added to the growth media: 30 µg/ml of nalidixic acid for *B. pertussis* wild type strain BP338 and its mutants; 30 µg/ml of gentamicin for *B. pertussis* mutants BP338BipA^−^, BP338Vag8^−^, 18–323Sm^r^Vag8^−^, RFBP2152, and BBC9BrkA; 100 µg/ml ampicillin for *E. coli* strains carrying pEG7-vag8, pTEN34 or pBluescript II KS^−^ (Stratagene) and derivatives; 34 µg/ml of chloramphenicol for *B. pertussis* or *E. coli* strains harboring pVag8 or pBrkA [16]); 50 µg/ml of streptomycin for 18–323Sm^r^ and its isogenic mutant 18–323Sm^r^Vag8^−^; 50 µg/ml of kanamycin for all *B. pertussis* transposon mutants, and BBC9BrkA, and *E. coli* strains harboring pBBR1MCS2 [36]. *B. pertussis* strains BP338, BP347and BBC9BrkA and the transposon mutants have been described previously [11], [15], [16]. The streptomycin-resistant 18–323 strain (18–323-Sm^r^) was isolated by plating 18–323 on streptomycin and picking a resistant colony. The plasmids pEG7 [37] and pTEN34 [38] were a gift from Peggy Cotter (University of North Carolina).

### Generation of *B. pertussis* Vag8 and BipA mutants

Suicide plasmid pTEN34 [38] was used to generate a BipA mutant of strain BP338. To generate *B. pertussis* Vag8 knock-out mutants, we constructed suicide vector pEG7-vag8 by PCR amplification of an internal fragment of *vag8* from genomic DNA of strain BP338 or 18–323-Sm^r^ using primers BPvag8fw3 (CGCGGATCCCGTCCGAGCACGGTATCAACG) and BPvag8rev3 (GGAATTCCACATAGATCCCGGCGACTTCC), and cloning of the PCR product into pEG7 [37] using BamHI and EcoRI (New England Biolabs). Suicide vectors were introduced into *B. pertussis* via conjugation using *E. coli* S17-1 as donor strain, and *B. pertussis* mutants were selected by growth on BG agar containing appropriate antibiotics (see above). Genomic integration of the suicide vectors into *bipA* or *vag8* was verified by PCR and sequencing of the generated products using pTEN34-specific primer lacZrev1 (TCGCACTCCAGCCAGCTTTC) and BipAfw2 (GTGCTGGCCGCAAGTCGAG); or pEG7fw1 (TAGGCGTATCACGAGGCCCTTTC) in combination with primer Vag8fw2 (CCCCAAGCTTCCAAGGCGTTTTCTGTCAATCG).

### Complementation of *vag8*

*vag8* was cloned downstream of the promoter region of *B. pertussis cpn10* in the pBBR1MCS vector backbone by swapping out the *brkA* gene from pBrkA [16]. The detailed construction of pBrkA [16]) is as follows. The entire *brkA* locus of vector pRF1066 [15], beginning from the NruI site of the adjacent *brkB* gene, was cloned into the broad-range vector pBBR1MCS using NruI and HindIII (New England Biolabs), and a 476-bp fragment of *brkB* was deleted using AatII (New England Biolabs). The promoter region of *cpn10* (P*_cpn10_*) was amplified by PCR from genomic DNA of *B. pertussis* BP338 (isolated with DNeasy® tissue kit from QIAGEN according to the manufacturer's instructions) using Vent® DNA polymerase (New England Biolabs) and primers Pcpn10fw1 (GTGTATCCCGGTACCTGAGCCCAGC) and Pcpn10rev1 (GACGCAGGTACCTGAGGAACTCCTG). To drive *brkA* expression, the PCR-amplified P*_cpn10_* sequence was cloned into the vector construct described above by use of KpnI (New England Biolabs). Correct P*_cpn10_* orientation was confirmed by restriction digest with SmaI (New England Biolabs).

pVag8 was cloned as follows. The *vag8* gene was amplified from genomic DNA of BP338 using Platinum Pfx DNA polymerase (Invitrogen) and primers BPvag8fw2 (CCCCAAGCTTCCAAGGCGTTTTCTGTCAATCG) and BPvag8rev2 (CGCTCTAGAGCATCGCCGTACCCTGAGC). Using the enzymes HindIII and XbaI (New England Biolabs), the amplified *vag8* gene was cloned into pBlueScript II KS^−^ (Stratagene) to generate the vector pBS*vag8*. pBrkA was digested with XhoI (New England Biolabs) and XbaI in order to remove the *brkA* gene from the vector, leaving the pBBR_P*cpn10*_ backbone. pBS*vag8* was digested with XhoI and XbaI and the released *vag8* fragment was cloned into the pBBR_P*cpn10*_ backbone producing the vector pVag8. The *vag8* gene in pVag8was sequenced and found to be identical to the Tohama-1 annotated sequence except for one silent mutation at base pair 246 (C to T).

The plasmids pBBR1MCS2 (empty vector), pBrkA and pVag8 were introduced into BP338Vag8KO via conjugation [11] or electroporation [39].

### Expression and purification of a histidine-tagged Vag8 passenger domain

A 1.7 kb fragment of DNA encoding the predicted passenger domain of Vag8 (residues Val^40^ to Leu^610^) was amplified by PCR from chromosomal DNA from BP338 using primers BPvag8fw8 (AAGGATCCGGTCACGGCAGCGCAGCG) and BPvag8rev7 (CCCCAAGCTTACAACTCGTTGGTCCGC), digested with BamHI and HindIII, and cloned into pET30b (Novagen). The resultant plasmid, pET30bVag8pass, was sequence-verified and transformed into BL21 (Novagen). A single transformant was picked, grown to an OD_600_ of approximately 0.6 and induced with isopropyl-B-D-thiogalactopyranoside (IPTG) at a final concentration of 1 mM. Expression of the Vag8 passenger was confirmed by immunoblot using an anti-His tag antibody (Santa Cruz Biotechnology). The histidine-tagged Vag8 passenger was purified under denaturing conditions using Ni-NTA using the procedure previously described to purify the BrkA passenger [26]. Urea was removed by dilution in order to promote folding of Vag8, and folding (acquisition of beta structure) was assessed by circular dichroism spectroscopy as described [26] at the Laboratory for Molecular Biophysics at the University of British Columbia.

### C1inh binding assays and other immunoanalyses

Freshly grown bacteria were harvested in pre-warmed (37°C) Stainer-Scholte (SS) broth without supplements [11]. To test the ability of viable *B. pertussis* cells to recruit C1inh from human serum, we employed a surface-binding assay that we developed [16]. In brief, bacteria were exposed to normal human serum for 15 min at 37°C, placed on ice for 5 min, washed 3 times and pelleted. Cell-associated C1inh was detected by immunoblot following SDS-PAGE. C1inh binding was also detected by a Far-Western or gel-overlay assay. Far-Western analysis was carried out similar to the gel overlay technique described by Gauthier and Finlay [24] with some modifications. Whole-cell lysates from 200-µL aliquots of bacteria at an OD_600_ of 0.25, or purified Vag8 or BrkA passenger domains were subject to 11% sodium dodecyl sulfate-polyacrylamide gel electrophoresis (SDS-PAGE) without prior serum incubation. Following SDS-PAGE, separated proteins were transferred onto an Immobilon-P membrane (Millipore) using 10 mM 3-(cyclohexylamino)-1-propanesulfonic acid (Research Organics) as transfer buffer (pH 11.0). Transfer was carried out at 30 V overnight or at 100 V for 1 h. Blots were washed three times for 20 min each in a 50 mM Tris buffer (pH 8.0) with 20% isopropanol, and then three times for 20 min each in 50 mM Tris buffer (pH 8.0). Subsequently, the blots were incubated two times for 30 min each in a buffer with 6 M guanidine-HCl and 50 mM Tris (pH 8.0), and then extensively washed for >18 h at 4°C in a pre-cooled wash buffer containing 0.05% Tween 20 and 50 mM Tris buffer (pH 8.0), with numerous changes of the wash buffer. Blots were then blocked with a 5% (w/v) skim milk solution in Tris-buffered saline with 0.1% Tween 20 (TBST) and overlaid with 1% normal human serum diluted in Veronal buffer (BioWhittacker), both for 1 h at room temperature. Subsequently, blots were washed six times for 5 min each in TBST, and probed with 1/10,000 dilutions of polyclonal goat anti-C1inh primary antibody (Calbiochem) and a 1/50,000 dilution of a horseradish peroxidase (HRP)-conjugated donkey anti-goat secondary antibody (Jackson ImmunoResearch Laboratories Inc.) as described [16], [40].

Standard immunoblot analysis was also used to test for BrkA expression in *B. pertussis* strains [16], [40] using a 1/30,000 dilution of a rabbit anti-rBrkA antiserum [41] and a 1/10,000 or 1/50,000 dilution of a HRP-conjugated goat anti-rabbit secondary antibody (Cappel, ICN Biomedicals) [16], [40]. Western Lightning chemiluminescence regent (Perkin Elmer) and X-OMAT blue autoradiography film (Kodak) were used for HRP detection.

To test C1inh binding by using ELISA, two-fold serial dilutions of purified, recombinant Vag8 or BrkA in PBS (pH 7.4) were prepared, with the top concentration at 5 µg/ml protein. Protein dilutions were used to coat Nunc MaxiSorp® flat-bottom 96 well plates over night at 4°C. All further steps were performed at room temperature. Plates were washed three times with PBS (pH 7.4) containing 0.05% Tween-20 (PBST) and non-specific binding was blocked by adding 50 µl 1× ELISA Diluent solution (eBioscience) to each well, followed by a 1 hr incubation. Following three washes with PBST, plates were incubated for 1 hr either with 10 µg/ml purified human C1inh (Advanced Research Technologies) in 1× ELISA Diluent solution or 2% normal human serum diluted in 1× Veronal buffer (BioWhittaker), as indicated. Plates were washed three times with PBST, and probed for 1 hr each with 2 µg/ml goat anti-C1inh antibody (Calbiochem) and 1 µg/ml HRP-conjugated donkey anti-goat secondary antibody (Jackson ImmunoResearch Laboratories Inc.) in 1× ELISA Diluent solution. After five final washes, HRP activity was measured using TMB substrate (eBioscience) and following a standard ELISA protocol.

### *B. pertussis* membrane enrichments and mass spectrometry analysis

Bacteria were grown on BG agar, harvested into, and washed twice with 10 mL of ice cold sucrose-Tris/HCl buffer (250 mM sucrose, 10 mM Tris, pH 7.0). After addition of EDTA to a final concentration of 1 mM and 1 tablet of Complete mini protease inhibitor cocktail (Roche), the cells were lysed by freeze-thawing using a dry-ice ethanol bath followed by pulsed sonication under constant cooling. The suspensions were centrifuged at 2000 *g* for 10 min at 4°C to pellet unbroken cells, and the supernatants were subject to ultracentrifugation at 100,000 *g* for 1 hour at 4°C to pellet the membrane fraction. Pellets were resuspended in 2.5 ml sucrose-Tris/HCl buffer and proteins were precipitated with trichloroacetic acid. Precipitated proteins washed with 1 ml ice-cold acetone and finally resuspended in 2 ml of sample buffer (7 M urea, 2 M thiourea, 4% w/v CHAPS, 0.5% v/v IPG buffer, 0.002% bromophenol blue, 18 mM dithiothreitol). The samples (40 µl) were then subjected to 11% SDS-PAGE, the polyacrylamide gel stained with Coomassie, and protein bands migrating at approximately 95 to 100 kDa were excised. Gel samples were analyzed by tandem mass spectrometry (MS/MS) at the MSL/LMB Proteomics Core Facility at the University of British Columbia. In brief, samples were subjected to reduction/alkylation with dithiothreitol/iodoacetamide followed by digestion with trypsin. The resulting peptides were desalted and concentrated with STAGE tips [42] and analyzed using a Thermo Electron LTQ-Orbitrap. Ions search of the obtained data against the MSDB database was performed using Mascot.

### Serum killing assays

Serum killing assays were performed as previously described [16] with the modification that viable bacteria were exposed to 10% normal or heat-inactivated human serum for 15 min ( 37°C) instead of 1 h using 96-well cell culture cluster (round bottom) polypropylene plates (Corning). The serum samples used in this study and elsewhere [17]were obtained from an adult [41], who had no recollection of exposure to *B. pertussis*. The percent survival was calculated by dividing the number of colony forming units (CFU) obtained after exposure of the bacteria to normal serum by the number of CFU obtained after exposure to heat-inactivated serum and multiplying this by 100. Unless otherwise noted, the experiments were done at least twice in triplicate. The Student's *t* test was used for statistical analysis.
